# Evaluation of the application of wild yeasts in inhibiting germination of ochratoxin-producing Fungi during coffee fermentation process

**DOI:** 10.1016/j.fochx.2024.102077

**Published:** 2024-12-09

**Authors:** Tsung-Yu Liu, Wei-Hsuan Hsu, Bao-Hong Lee

**Affiliations:** a Department of Food Safety/Hygiene and Risk Management, College of Medicine, National Cheng Kung University, Tainan 701401, Taiwan; b Department of Horticultural Science, National Chiayi University, Chiayi 60004, Taiwan

**Keywords:** Coffee, Ochratoxin, *Aspergillus niger*, *Saccharomyces cerevisiae*, Citric acid

## Abstract

Specialty coffee, typically lightly roasted, is valued for its unique fruity aroma. However, the fermentation process poses a risk of contamination with ochratoxin-producing fungi. This study aimed to select wild yeast strains capable of contributing distinctive flavor profiles while inhibiting the growth of ochratoxin-producing fungi. Coffee pulp served as a substrate to simulate yeast growth during coffee fermentation, allowing for the evaluation of yeast metabolites potential to inhibit spore germination in ochratoxin-producing fungi (*Aspergillus niger*). The findings demonstrated that the *Saccharomyces cerevisiae* strain effectively inhibited spore germination in *A. niger*. High-performance liquid chromatography (HPLC) analysis indicated that citric acid is likely the primary organic acid responsible for inhibiting *A. niger* spore germination. These results suggested that *S. cerevisiae* has potential applications in enhancing the food safety of coffee.

## Highlights

• Wild yeast strains capable of contributing distinctive flavor.
• *Saccharomyces cerevisiae* potentially inhibited spore germination in *Aspergillus niger.*
• *S. cerevisiae* has potential applications in enhancing the food safety of coffee.

## 1 Introduction

Ochratoxin is a common contaminant found in various foods, including coffee, wine, beer, grains, spices, cocoa products, and grape juice (Bryden, 2007). In Europe, exposure to ochratoxin from coffee products accounts for approximately 8.1 % of cases (Annunziata et al., 2024). Ochratoxins are classified into three types: A, B, and C, with ochratoxin A (OTA) being the most toxic. The International Agency for Research on Cancer (IARC) has classified OTA as a Group 2B carcinogen, indicating it is possibly carcinogenic to humans. Since the 1970s, OTA has been implicated in various human renal diseases, including Balkan Endemic Nephropathy (BEN) (Castegnaro et al., 2006) and Chronic Interstitial Nephropathy (CIN) (Abid et al., 2003; Bui-Klimke & Wu, 2015). OTA is a low molecular weight secondary metabolite produced by molds and is characterized as a weak organic acid. Structurally, it is formed by a peptide bond linkage between phenylalanine and dihydro-isocoumarin (Leitão, 2019).

Fungal contamination of coffee and the subsequent production of ochratoxin can be influenced by numerous factors, including fungal strain type, water activity, climatic conditions, storage, transportation, and processing methods (Napolitano et al., 2007; Batista et al., 2003; **Bucheli & Taniwaki,** 2002; **Paterson & Lima,** 2010). Studies have shown that brewing coffee is ineffective in removing OTA (Studer-Rohr et al., 1995; Tsubouchi et al., 1988). However, OTA can be degraded during coffee roasting, although the extent of degradation depends on the roasting temperature, initial OTA concentration, and contaminating fungal strains (Nehad et al., 2005; Studer-Rohr et al., 1995).

Molds, a category of fungi, are eukaryotic microorganisms recognized for forming hyphae but lacking fruiting bodies, which classifies them as filamentous fungi. They thrive particularly well in warm, humid environments, with optimal growth occurring at temperatures between 10 °C and 40 °C and requiring a water activity level of 0.85 or above. As obligate aerobes, molds depend on oxygen to sustain their growth, reproduction, and metabolism (**Lima et al., 2022**). Natural fermentation that occurs through the interplay of various yeasts, lactic acid bacteria, acetic acid bacteria, aerobic bacteria, and a range of filamentous fungi (**Guzmán-Alvarez & Márquez-Ramos,** 2021).

The coffee cherry is composed of several distinct layers from the outer to the inner portions: the outer skin, pulp, pectin layer, parchment, silver skin, coffee bean, and center cut (**Cruz-O'Byrne et al., 2020**). Coffee pulp, the primary byproduct generated during coffee processing, accounts for 29 % of the coffee cherry's dry weight and approximately half of its wet weight in washed processing methods, presenting a significant challenge for coffee production. This study aimed to select wild yeast strains with the potential to inhibit spore germination of ochratoxin-producing fungi. Drawing on recent advances in anaerobic fermentation techniques (**Lee, Huang, et al., 2023**), we utilized coffee pulp as a substrate to simulate the anaerobic fermentation process of coffee and analyzed the metabolites produced by these microorganisms.

## 2 Materials and methods

### 2.1 Wild yeast isolation and identification

Fresh cocoa beans were fermented under anaerobic conditions, and microbial strains were subsequently isolated. The initial step in microbial identification involved assessing colony morphology. Polymerase Chain Reaction (PCR) was employed to amplify the 18S rDNA gene fragment. The size of the PCR products was confirmed through gel electrophoresis, followed by DNA purification using a PCR clean-up kit (HiYield™ Gel/PCR Fragments Extraction Kit). The DNA concentration was measured with a Nanodrop spectrophotometer. The purified DNA was then submitted to the Genome Medicine Center at National Cheng Kung University for nucleotide sequencing using Sanger sequencing. This was conducted on the ABI 3130xl DNA sequencer, utilizing the third-generation ABI BigDye Terminator reagent (Version 3.1). The resulting gene sequences were compared against the NCBI database (https://www.ncbi.nlm.nih.gov/) to confirm the microbial species, which included *Candida glabrata* (YF342), *C. glabrata* (YB761), *C. glabrata* (ME541), *Pichia kudriazevii* (YB361), *P. kudriazevii* (YB661), *P. kudriazevii* (MF841), *P. kudriazevii* (MC741), *P. kudriazevii* (MB842), *Pichia barkeri* (Y24V1), and *Saccharomyces cerevisiae* (Y32X1). The strains of *S. cerevisiae* (BCRC 21992) and *Torulaspora delbrueckii* (BCRC 21429) were obtained from the Bioresource Collection and Research Center (Hsinchu, Taiwan).

### 2.2 Growth growth of yeast in the coffee pulp

To determine the appropriate inoculum levels for subsequent applications in coffee cherry and coffee pulp fermentation, growth curves of various strains were analyzed to assess the relationship between cultiure time and microbial density. The strains tested included *C. glabrata* (YF342), *C. glabrata* (YB761), *P. kudriazevii* (YB361), *P. kudriazevii* (YB661), *P. kudriazevii* (MF841), *P. kudriazevii* (MC741), *C. glabrata* (ME541), *P. kudriazevii* (MB842), *S. cerevisiae* (BCRC 21992), *P. barkeri* (Y24V1), *S. cerevisiae* (Y32X1), and *T. delbrueckii* (BCRC 21429). The growth curves (triplicate) of various microbes were evaluated by optical density (OD) measurements at 600 nm. The resulting optical density and microbial count data were analyzed and graphed using GraphPad Prism 8 software (version 8.0.1).

### 2.3 Coffee pulp fermentation

After depulping the coffee cherries using a depulping machine, the coffee pulp was collected for further use. One hundred grams of coffee pulp were combined with 30 g of deionized water and homogenized using a blender, followed by sterilization in an autoclave at 121 °C for 15 min. Following sterilization, the pulp was inoculated with various microorganisms, including *C. glabrata* (YF342), *C. glabrata* (YB761), *P. kudriazevii* (YB361), *P. kudriazevii* (YB661), *P. kudriazevii* (MF841), *P. kudriazevii* (MC741), *C. glabrata* (ME541), *P. kudriazevii* (MB842), *S. cerevisiae* (BCRC 21992), *P. barkeri* (Y24V1), *S. cerevisiae* (Y32X1), *T. delbrueckii* (BCRC 21429), and a control group (no inoculum). The fermentation was carried out at 25 °C for five days. During the cultivation period, 100 μL samples of the fermentation broth were plated daily to monitor microbial growth. To maintain anaerobic conditions, the anaerobic packs were replaced daily during the sampling process. After fermentation was complete, the fermentation broth was collected for subsequent experiments.

### 2.4 Preparation of spore obtained from *Aspergillus niger* BCRC 33485

The microorganism used in this experiment is Aspergillus niger BCRC 33485 (Bioresource Collection and Research Center, Hsinchu, Taiwan), a strain known for its ability to produce OTA. The fungus was cultured on Potato Dextrose Agar (PDA). Once mycelia had developed, a sterilized knife and tweezers were used to cut out 0.5 cm × 0.5 cm squares from the edge of the mycelial mat. These mycelial pieces were placed in 1.5 mL microcentrifuge tubes containing 0.5 mL of glycerol and 0.5 mL of Potato Dextrose Broth (PDB). Following incubation, the tubes were transferred to a − 80 °C freezer for long-term storage (Nakasone et al., 2004).

To prepare a spore suspension, the filamentous fungus was cultured on PDA until spore formation was observed. The spores were then dislodged using a sterilized spatula or sterilized applicator stick in PDB. The resulting suspension was collected into centrifuge tubes and centrifuged at 10,000*g* for 10 min at 4 °C to break up the mycelia. Subsequently, the suspension was filtered through sterile Miracloth to isolate single spores. After resuspension in a buffer solution and appropriate dilution, a 10 μL aliquot was placed on a hemocytometer and observed under a microscope for counting (Sinnelä et al., 2021).

### 2.5 Spore germination inhibition test using coffee pulp fermented products

After preparing the spore suspension, the number of spores were counted using a hemocytometer under an optical microscope. For each plate, ten spores were spread for cultured to compare germination rates. The fermented coffee pulp liquid was filtered through a 0.22 μm membrane to remove fermentation microorganisms, and then mixed with the spore suspension at a 4:1 ratio. The mixture is incubated at 25 °C for two hours with shaking on a shaker. Subsequently, the mixture (100 μL) was spread onto PDA for culture, with the procedure repeated in triplicate. After three days of incubation at 25 °C, the number of spore germinations is counted to assess whether the metabolites in the different fermentation liquid samples have inhibitory effects on spore germination.

### 2.6 Organic acid analysis of coffee pulp fermented product

For the organic acid analysis, samples were collected following the fermentation of coffee husks and were analyzed for several organic acids, including succinic acid, fumaric acid, oxalic acid, citric acid, tartaric acid, malic acid, acetic acid, and lactic acid. The analytical column utilized was a stainless-steel column measuring 25 cm in length and 4.6 mm in internal diameter, packed with amine-modified silica with a particle size of 5–7 μm. Chromatography was performed using a mobile phase consisting of acetonitrile and deionized water (80:20, *V*/V) at a flow rate of 1.3 mL per minute. Detection was accomplished using a Refractive Index Detector (Waters® TaperSlit, USA), maintained at a temperature of 30 °C.

### 2.7 Statistical analysis

The experimental results were obtained from triplicate analyses and are presented as the mean ± standard deviation (SD). Data analysis was performed using the Statistical Analysis System (SAS Inc., Cary, NC, USA). One-way analysis of variance (ANOVA) was conducted to assess differences among groups, and Duncan's multiple range test was used to determine significant differences between means. A *p*-value of less than 0.05 was considered statistically significant.

## 3 Results

### 3.1 Wild yeast isolation and identification

Microorganisms for coffee pulp fermentation were selected from fermented cocoa beans and cocoa pods. Samples were collected at two, three, five, and seven days of fermentation and were plated on YPD and MRS media to isolate the microorganisms. Additionally, coffee fermentation starter cultures were selected using the same microbial screening method. A total of 216 single microbial strains were isolated. These strains were cultured on agar plates, and once the microorganisms had colonized the plates, the lids were slightly opened to assess the aromas through sniffing.

As summarized in Table 1, indicated that 55 strains exhibited floral aromas, (Eight strains exhibiting fruity aromas were selected for sequencing) identified as MF841, MC741, MB842, ME541, YB361, YB761, YB661, and YF342. Additionally, from the commercial coffee fermentation starter cultures, 20 strains were selected, with two strains, Y24V1 and Y32X1, chosen for sequencing.

**Table 1:** Isolated-microbial strains and special odor characteristics.

Number	Odor	Number	Odor	Number	Odor	Number	Odor
YA161		YF242		MB161		MA442	
YA181	II	YF142	I	MF141	II	MA361	
YA281		YF143	I	MF143		MA341	I
YB241	I	YF241		MF142		MA342	
YB261	I	YF261	I	MF241	I	MA443	
YB262	I	YF141		MF243		MA441	I
YB161	I	YF262		MF242		MB462	
YB162		YF161	I	MG242		MB363	
YB163		YG241	I	MG161		MB346	
YC241	V	YG142		MG262		MB362	
YD182	IV	YG141	I	MG141		MB441	I
YD181		YG242		MG142		MB341	
YD261		YG161		MG261		MB344	
YD161	III	YG261		MG281		MB342	II
YD162	IV	MA281		YB361	I	MB361	
YD141	IV	MA162		YF344		MB343	II
YD142	III	MB282		YF343		MB442	II
YE242		MB281		YF462		YD361	III
YE241		MB261	II	YG441		YF362	
YE281		MB142		YG341		YF442	
YE261		MB141		YG442		YF461	I
YA341	I	YC441		MG442		YF441	I
YA342	I	YC341	I	MG461		YF443	
YA361	I	YD441		MG361		YF342	I

YC841	I	YD761	II	YG841	III	YA742	III
YF742	III	YD741		YA741	I	YA861	III
YA561	I	YD542		MF361		YC641	
YA661	III	YF641		MF342		YD661	
YA681	III	YF661		MF441	I	YD642	
YB661	I	YF542		MB461	I	YD641	
YB641		YF642		MA561	I	YD541	
YB561		YF541	I	MA641		MB542	
YB541	I	YG561		MA541	I	MB641	
YC642		YG542		MA461		MC662	
YC561		YG541	I	MB543	I	MC661	II
YC542	I	YG641	I	MB643	I	MF561	
YC541	I	YC843	III	YF842	III	YA841	I
YD861	I	YD841	I	YF741	I	YA743	III
YE741	I	YD742	I	YG741		YA761	I
YA461	III	YD362		MF343		YC341	I
YA443	I	YD461		MF341		YC643	
YF841	I	YB862	I	MB444		MD442	
YC761	I	YB841	I	MB443	III	ME341	
YC741	I	YB761	I	MD462		MF442	
MD561		YB861	I	MD461		MF444	
ME541	I	YB762	I	MD481		ME342	

MD681		YB343		MD361		YB462	I
YB442		YB441	I	YB461		YB481	I
MC741	I	YF361	I	MF841	II	MB842	I
YA441	III	YD363		MG341		YF341	
YA481	III	YD442		MF443		YC441	
ME542	II	YB741	I	MD341		ME361	
MF544		YC742	I	MD342		MG441	
MF542		YC841	I	MD441		MB444	
YB362		YB341	I	YB361	I	YB363	I

Each flavor is represented by a Roman numeral code: I denotes floral notes; II denotes fermented fruit notes; III denotes smoky or dried squid notes; IV denotes rice cracker (biscuit) notes; and V denotes papaya seed notes.

**Fig. 1:**
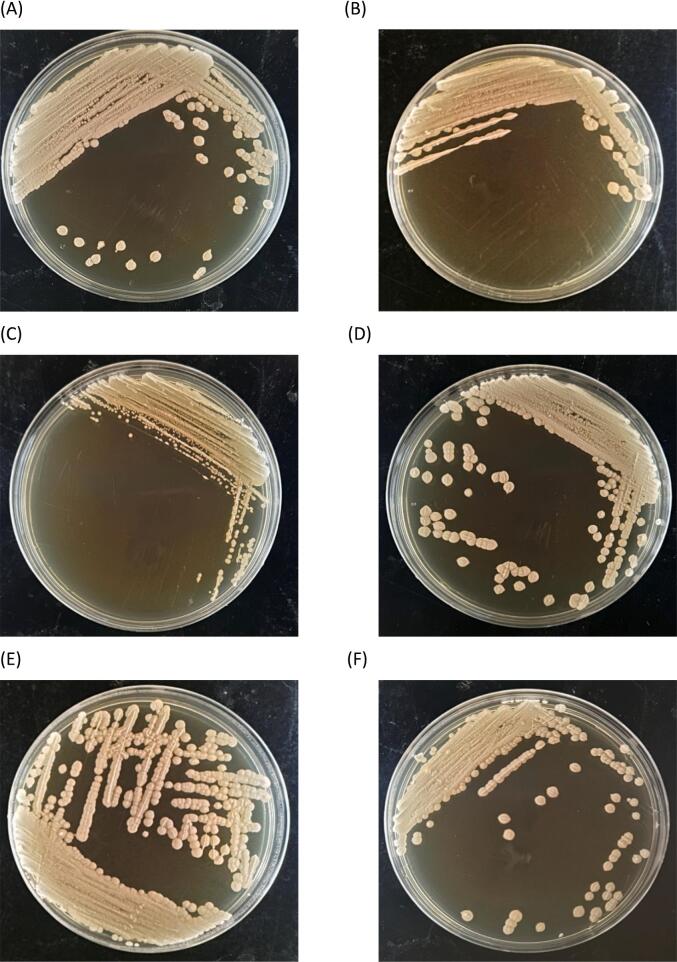

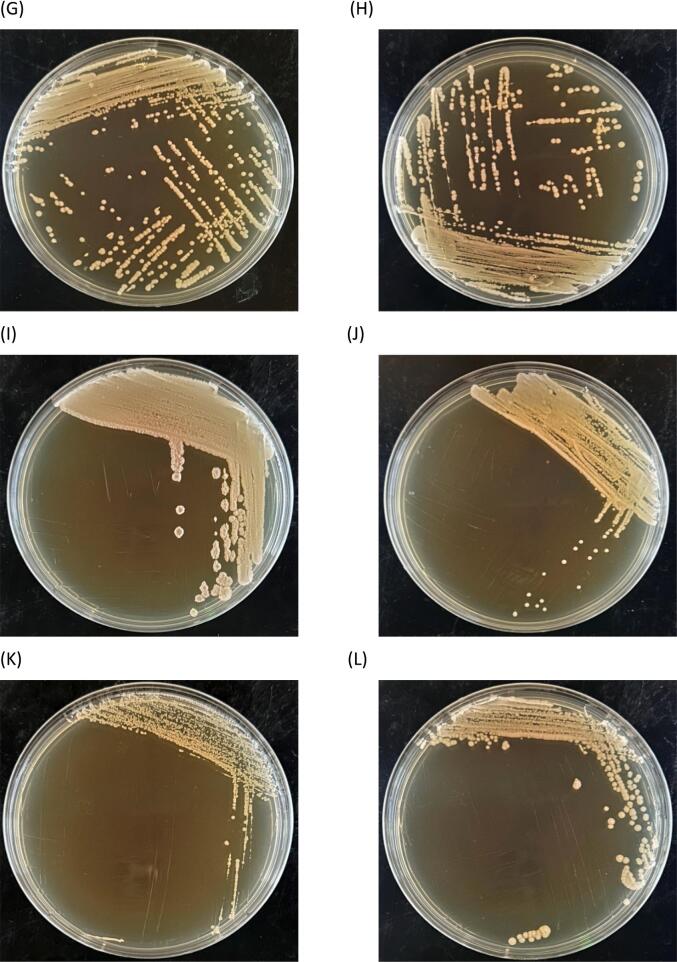
Diagram of yeast colony morphology. (A) MB842; (B) MC741; (C) ME541; (D) MF841; (E) YB361; (F) YB661; (G) YB761; (H) YF342; (I) Y24V1; (J) Y32X1; (K) SC21992; (L) TD.

DNA electrophoresis confirmed that the PCR-amplified 18S rDNA fragments were 1.1 kilobases (kb) in size. Sanger sequencing and comparison with the NCBI (National Center for Biotechnology Information) database revealed the following identifications: MF841, MC741, MF842, YB661, and YB361 as *P. kudriavzevii*; YB761, YF342, and ME541 as *C. glabrata*; Y24V1 as *P. barkeri*; and Y32X1 as *S. cerevisiae.* The results of the microbial identification are provided in Table 2. The colony morphologies of the 12 microbial strains were illustrated in Fig. 1.

**Table 2:** Identification of wild yeast strain.

Isolation name	Scientific name	Description
YB661	*Pichia kudriavzevii*	*Pichia kudriavzevii* NRRL Y-5396
YB361	*Pichia kudriavzevii*	*Pichia kudriavzevii* NRRL Y-5396
YB761	*Candida glabrata*	*Candida glabrata* NRRL Y-65
YF342	*Candida glabrata*	*Candida glabrata* NRRL Y-65
MF841	*Pichia kudriavzevii*	*Pichia kudriavzevii* NRRL Y-5396
MC741	*Pichia kudriavzevii*	*Pichia kudriavzevii* NRRL Y-5396
MB842	*Pichia kudriavzevii*	*Pichia kudriavzevii* NRRL Y-5396
ME541	*Candida glabrata*	*Candida glabrata* NRRL Y-65
Y24V1	*Pichia barkeri*	*Pichia barkeri* NRRL Y-17350
Y32X1	*Saccharomyces cerevisiae*	*Saccharomyces cerevisiae* NRRL Y-12632

### 3.2 Growth condition of yeast in the coffee pulp

To assess the growth and adaptability of yeast strains isolated from cocoa beans for coffee fermentation, we conducted a five-day fermentation experiment using coffee pulp. Daily sampling and plate counting were performed to monitor microbial growth. *P. kudriazevii*-YB661 started with an initial concentration of 4.978 ± 0.170 (log CFU/g). After one day, the microbial count increased to 7.795 ± 0.004 (log CFU/g), representing a growth factor of approximately 692-fold. The count remained relatively stable from days two through five, indicating that *P. kudriazevii*-YB661 exhibits good growth and stability in coffee pulp. *P. kudriazevi*i-YB361 had an initial concentration of 4.857 ± 0.074 (log CFU/g). After one day, the count surged to 8.747 ± 0.008 (log CFU/g), yielding a growth factor of about 7846-fold. Although there was a slight decrease to 7.968 ± 0.059 (log CFU/g) on day two, the count remained stable from days three through five. This strain demonstrated excellent growth, particularly with very high counts on day one. *P. kudriazevii*-MF841 had an initial concentration of 5.361 ± 0.041 (log CFU/g). After one day, it increased to 8.377 ± 0.026 (log CFU/g), a growth factor of about 1045-fold. The count peaked at 8.633 ± 0.015 (log CFU/g) on day two, representing a 1890-fold increase, and remained high through day five. *P. kudriazevii*-MC741 started with 5.712 ± 0.157 (log CFU/g). After one day, the count increased to 8.483 ± 0.070 (log CFU/g), a growth factor of 613-fold. It reached a peak of 8.821 ± 0.093 (log CFU/g) on day two, a 1384-fold increase, and remained high through day five. *P. kudriazevii*-MB842 initially had 5.805 ± 0.027 (log CFU/g). After one day, it increased to 8.223 ± 0.163 (log CFU/g), reflecting a growth factor of 278-fold. The count remained relatively stable until day four, when it peaked at 8.630 ± 0.065 (log CFU/g), a 675-fold increase.

*C. glabrata*-YB761 initially had a concentration of 5.397 ± 0.048 (log CFU/g). After one day, it increased to 8.223 ± 0.064 (log CFU/g), a growth factor of approximately 683-fold. By day two, the count reached 8.431 ± 0.095 (log CFU/g), representing a 1094-fold increase from the initial count. The count remained high up to day five, indicating consistent growth. *C. glabrata*-YF342 started with 6.411 ± 0.016 (log CFU/g). After one day, the count reached 8.524 ± 0.059 (log CFU/g), reflecting a 130-fold increase. The count peaked at 8.771 ± 0.076 (log CFU/g) on day three, achieving a 231-fold increase from the initial count, and remained stable through day five. This strain also showed good growth. *C. glabrata*-ME541 began with 5.324 ± 0.073 (log CFU/g). After one day, the count reached 8.194 ± 0.021 (log CFU/g), a 747-fold increase. It peaked at 8.805 ± 0.186 (log CFU/g) on day two, representing a 3380-fold increase, and maintained this high level until day five.

*P. barkeri*-Y24V1 had an initial concentration of 4.339 ± 0.260 (log CFU/g). After one day, the count increased to 7.388 ± 0.102 (log CFU/g), a growth factor of 1133-fold. By day three, the count reached 7.760 ± 0.022 (log CFU/g), a 2636-fold increase, and remained stable through day five. However, this strain exhibited a lower peak count compared to others.

*S. cerevisiae*-Y32X1 had a growth factor of about 711-fold. The count reached 8.118 ± 0.046 (log CFU/g) on day two, a 921-fold increase from the initial count, indicating good growth. *S. cerevisiae* BCRC 21992 (SC21992) started with 5.022 ± 0.019 (log CFU/g). After one day, it increased to 8.589 ± 0.049 (log CFU/g), a 3703-fold increase. However, the count declined to 7.113 ± 0.020 (log CFU/g) by day five, indicating that while initial growth was high, it decreased substantially over time. *T. delbrueckii* (TD) began with 4.632 ± 0.027 (log CFU/g). After one day, the count increased to 7.230 ± 0.115 (log CFU/g), a 407-fold increase. It peaked at 7.940 ± 0.084 (log CFU/g) on day four, representing a 1590-fold increase, but decreased slightly by day five. This strain also demonstrated good growth, though its peak counts were lower compared to other strains. In summary, the growth data reveal that *P. kudriazevii*-YB361 exhibited the highest growth factor after one day, while *C. glabrata*-ME541 had the highest growth factor from days two through five. Peak microbial counts generally ranged between 8.0 and 8.9 log CFU/g, with *P. barkeri*-Y24V1, *S. cerevisiae*-BCRC 21992, and *T. delbrueckii* (TD) showing lower peak counts compared to other strains. Overall, yeasts isolated from cocoa beans demonstrated superior growth in coffee pulp compared to commercial and standard strains.

### 3.3 Spore germination inhibition test by coffee pulp fermented product

**Fig. 2:**
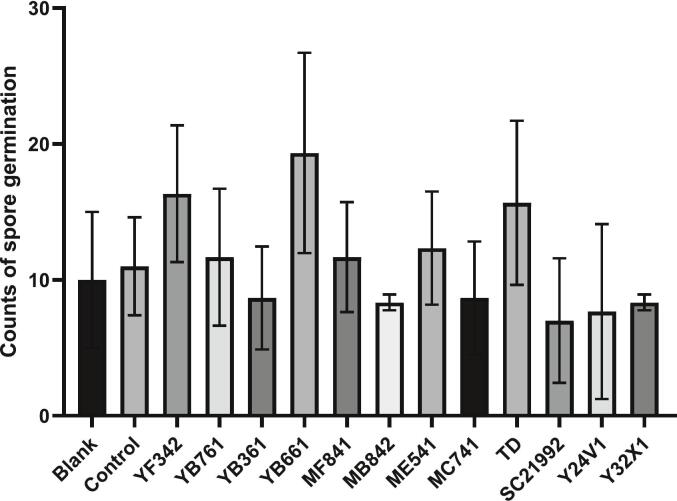
Result of coffee pulp fermented metabolites by different microorganisms on *Aspergillus niger* BCRC 33485 spore germination inhibition.

After preparing the coffee pulp fermentation liquid, it was co-cultured with a spore suspension of *Aspergillus niger* BCRC 33485 to observe spore germination. The results are summarized in Table 3 and Fig. 2. The blank group represents the spore suspension alone, while the control group refers to the coffee pulp fermentation liquid without microbial inoculation. The findings indicated that the spore germination counts were 10.00 ± 5.00 for the blank group and 11.00 ± 3.60 for the control group, suggesting that the metabolites from the coffee pulp, when fermented anaerobically for five days, do not inhibit spore germination and may even promote it in the absence of microbial inoculation. Among the twelve microbial fermentation groups, four demonstrated a notable ability to inhibit spore germination effectively: *S. cerevisiae-*BCRC 21992, with a spore germination count of 7.00 ± 4.28 (spores/plate); *S. cerevisiae*-Y32X1, with a spore germination count of 8.33 ± 0.57 (spores/plate); *P. kudriavzevii*-MB842, also with a spore germination count of 8.33 ± 0.57 (spores/plate); and *P. kudriavzevii*-MC741, with a spore germination count of 8.67 ± 4.16 (spores/plate).

**Table 3:** Spore germinates inhibition degree of coffee pulp fermented product.

Group	Particle of spore germination per plate
Spore suspension (Blank)	10.00 ± 5.00
Control	11.00 ± 3.60
YF342 (*Candida glabrata*)	16.33 ± 5.03
YB761 (*Candida glabrata*)	11.67 ± 5.03
YB361 (*Pichia kudriavzevii*)	12.00 ± 5.56
YB661 (*Pichia kudriavzevii*)	19.33 ± 7.37
MF841 (*Pichia kudriavzevii*)	11.67 ± 4.04
MB842 (*Pichia kudriavzevii*)	8.33 ± 0.57
ME541 (*Candida glabrata*)	12.33 ± 4.16
MC741 (*Pichia kudriavzevii*)	8.67 ± 4.16
*Torulaspora delbrueckii* BCRC 21429	15.67 ± 6.02
*Saccharomyces cerevisiae* BCRC 21992	7.00 ± 4.28
Y24V1 (*Pichia barkeri*)	11.00 ± 5.29
Y32X1 (*Saccharomyces cerevisiae*)	8.33 ± 0.57

The data were obtained from triplicate experiments, and the results are presented as mean ± standard deviation (n = 3). Significant difference was shown by various letters (*p* < 0.05).

These results indicated that the *S. cerevisiae* strains exhibit a greater ability to inhibit spore germination compared to other strains. Conversely, *C. glabrata* and *P. kudriavzevii* strains did not show significant inhibitory effects on spore germination.

### 3.4 Organic acid analysis of coffee pulp fermented product

In the aforementioned experiment, the organic acid composition and concentration of four spore-inhibiting groups, along with the control group, were analyzed to investigate their relationship with spore germination. The results are presented in Table 4. The control group had a total organic acid concentration of 1.14 g/100 mL, comprising succinic acid (0.17 g/100 mL), acetic acid (0.16 g/100 mL), and lactic acid (0.81 g/100 mL). The *S. cerevisiae*-BCRC 21992 group had a total organic acid concentration of 1.06 g/100 mL, consisting of succinic acid (0.16 g/100 mL), citric acid (0.09 g/100 mL), acetic acid (0.12 g/100 mL), and lactic acid (0.69 g/100 mL). The *S. cerevisiae*-Y32X1 group exhibited a total organic acid concentration of 1.00 g/100 mL, which included succinic acid (0.18 g/100 mL), citric acid (0.09 g/100 mL), malic acid (0.10 g/100 mL), acetic acid (0.11 g/100 mL), and lactic acid (0.52 g/100 mL). The *P. kudriavzevii*-MB842 group showed a total organic acid concentration of 0.75 g/100 mL, with succinic acid (0.17 g/100 mL), citric acid (0.08 g/100 mL), acetic acid (0.12 g/100 mL), and lactic acid (0.38 g/100 mL). The *P. kudriavzevii*-MC741 group had a total organic acid concentration of 0.87 g/100 mL, comprising succinic acid (0.19 g/100 mL), citric acid (0.09 g/100 mL), acetic acid (0.13 g/100 mL), and lactic acid (0.46 g/100 mL).

**Table 4:** Result of organic acid content of coffee pulp fermented liquid.

	Control	MC741 (*Pichia kudriavzevii*)	MB842 (*Pichia kudriavzevii*)	Y32X1 (*Saccharomyces cerevisiae*)	SC21992 (*Saccharomyces cerevisiae*)
	(g/100 mL)				
Succinic acid	0.17 ± 0.01^b^	0.19 ± 0.01^b^	0.17 ± 0.04^b^	0.18 ± 0.01^b^	0.16 ± 0.02^b^
Fumaric acid	–	–	–	–	–
Oxalic acid	–	–	–	–	–
Citric acid	–	0.09 ± 0.02^c^	0.08 ± 0.01^c^	0.09 ± 0.00^c^	0.09 ± 0.00^c^
Malic acid	–	–	–	0.10 ± 0.00^c^	–
Acetic acid	0.16 ± 0.01^b^	0.13 ± 0.01^bc^	0.12 ± 0.03^b^	0.11 ± 0.00^c^	0.12 ± 0.01^bc^
Lactic acid	0.81 ± 0.03^a^	0.46 ± 0.01^a^	0.38 ± 0.05^a^	0.52 ± 0.01^a^	0.69 ± 0.03^a^
Tartaric acid	–	–	–	–	–

The data were obtained from triplicate experiments, and the results are presented as mean ± standard deviation (*n* = 3). Significant difference was shown by various letters (*p* < 0.05).

The results indicate that the concentrations of succinic acid and acetic acid among the five groups are relatively similar, suggesting that the presence or absence of microbial inoculation and the type of strain inoculated have minimal impact on these two organic acids. Notably, the *S. cerevisiae*-Y32X1 group contained 0.10 g/100 mL of malic acid, which was absent in the other inoculated groups. The lactic acid content ranged from 0.38 to 0.81 g/100 mL across the groups, with the highest concentration observed in the control group, decreasing following microbial inoculation. Citric acid was only detected in the inoculated groups, with concentrations ranging from 0.08 to 0.09 g/100 mL. Given that these four inoculated groups exhibited spore germination inhibitory effects, it is hypothesized that the presence of citric acid may be strongly correlated with the inhibition of spore germination.

### 3.5 The pH level detection of coffee pulp fermented product

**Fig. 3:**
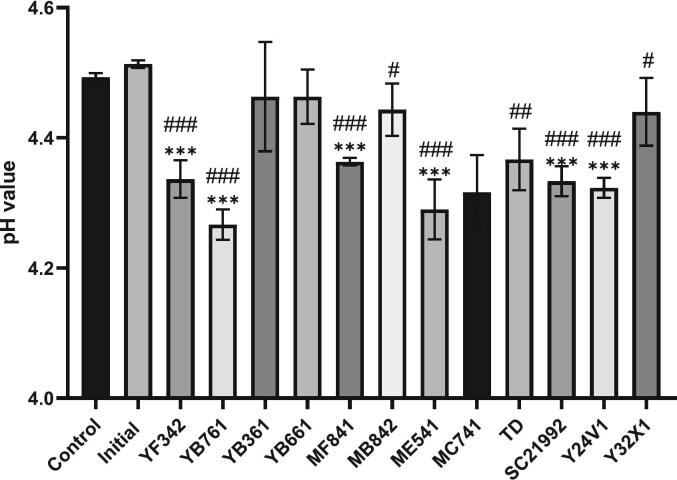
Comparison of pH level of different microorganisms in coffee pulp fermentation. The data were obtained from triplicate experiments, and the results are presented as mean ± standard deviation (*n* = 3). Significant difference was shown by various letters (*p* < 0.05).

The pH measurements were taken at the beginning of inoculation and at the end of the fifth day. The initial pH value was 4.51 ± 0.01, while the results after five days are presented in Table 5 and Fig. 3. The data reveal that the pH of the control group exhibited minimal change, remaining at 4.49 ± 0.01. Among the microbial inoculated groups, the smallest decrease in pH was observed in the *P. kudriavzevii*-YB361 and -YB661 groups, with pH values of 4.46 ± 0.04 and 4.46 ± 0.01, respectively. In contrast, the greatest pH reduction was recorded for the *C. glabrata-*YB761 and -ME541 groups, with pH values of 4.27 ± 0.08 and 4.29 ± 0.05, respectively. These results suggested that *C. glabrata* exhibits a greater capacity for pH reduction compared to *P. kudriavzevii*. Although *S. cerevisiae*-Y32X1 and *S. cerevisiae*-BCRC 21992 are both strains of *S. cerevisiae*, their pH values differed, measuring 4.44 ± 0.05 and 4.33 ± 0.02, respectively, indicating distinct properties between the two strains.

**Table 5:** PH level of coffee pulp before and after fermentation.

Group	pH level
Control	4.49 ± 0.01
YF342 (*C. glabrata*)	4.34 ± 0.02
YB761 (*C. glabrata*)	4.27 ± 0.08
YB361 (*P. kudriavzevii*)	4.46 ± 0.04
YB661 (*P. kudriavzevii*)	4.46 ± 0.01
MF841 (*P. kudriavzevii*)	4.36 ± 0.04
MB842 (*P. kudriavzevii*)	4.44 ± 0.05
ME541 (*C. glabrata*)	4.29 ± 0.05
MC741 (*P. kudriavzevii*)	4.32 ± 0.06
*T. delbrueckii* BCRC 21429	4.37 ± 0.05
*S. cerevisiae* BCRC 21992	4.33 ± 0.02
Y24V1 (*P. barkeri*)	4.32 ± 0.02
Y32X1 (*S. cerevisiae*)	4.44 ± 0.05

The data were obtained from triplicate experiments, and the results are presented as mean ± standard deviation (n = 3).

## 4 Discussion

The coffee cherry, the fruit of the coffee tree, is rich in carbohydrates, proteins, and minerals, and contains significant amounts of tannins, polyphenols, and caffeine (Bressani et al., 1972). The organic components of the coffee cherry include tannins (1.80–8.56 %), total pectic substances (6.5 %), reducing sugars (12.4 %), non-reducing sugars (2.0 %), caffeine (1.3 %), chlorogenic acid (2.6 %), and total caffeic acid (1.6 %) (**Murthy & Naidu,** 2012). The mucilage layer is primarily composed of carbohydrates, including polysaccharides such as cellulose and pectin, as well as monosaccharides such as glucose, mannose, xylose, arabinose, galactose, fructose, rhamnose, and uronic acid (Avallone et al., 2001).

The primary objective of coffee fermentation is to detach the mucilage layer from the parchment. Several conventional methods, including the dry process, are utilized to physically remove the hull after sun drying; however, in regions with limited sunlight, prolonged drying periods can lead to fungal contamination of the coffee beans (Silva et al., 2008). As a result, wet processing techniques that rely on microbial breakdown to eliminate the mucilage layer have been developed in areas with abundant water resources. During microbial metabolism in the mucilage layer, the metabolic byproducts of microbes contribute to the formation of precursors and flavor compounds in coffee, yielding a product with floral and fruity notes (Batista et al., 2009; Evangelista et al., 2015). By carefully controlling fermentation conditions to favor the growth of beneficial microorganisms, it is possible to inhibit the proliferation of potentially harmful fungi while enhancing the development of the product primary flavor characteristics (**Payne & Bruce,** 2001; Shi et al., 2024). Therefore, this study utilized wild yeasts isolated from cocoa beans (Table 1, Table 2; Fig. 2) and applied strains that enhance fermentation flavor to the fermentation of coffee.

Common microorganisms that produce ochratoxin A (OTA) include species from the genera *Aspergillus* and *Penicillium*, with *Aspergillus ochraceus*, *Aspergillus carbonarius*, *Aspergillus niger*, and *Penicillium verrucosum* being the primary contributors. These fungi have optimal growth temperatures ranging from 0 °C to 40 °C, with *A. niger* exhibiting the broadest range, from 6 °C to 47 °C. OTA has been shown to exhibit various toxic effects, particularly nephrotoxicity (Krogh, 1992), and has also been associated with hepatotoxicity, teratogenicity, carcinogenicity, and immunosuppressive properties (Bendele et al., 1985; Chopra et al., 2010; Haubeck et al., 1981; Mayura et al., 1976). Previous studies have investigated the inhibitory effects of lactic acid bacteria metabolites on fungal growth, revealing a strong correlation between the composition and concentration of organic acids and the inhibition of *Aspergillus* species (Bukhari et al., 2020; Shi et al., 2022). To gain a deeper understanding of the mechanisms underlying the observed spore inhibition, an analysis of organic acids in the effective inhibition groups was conducted. Food preservation can be achieved through various methods, including the creation of environments that are unfavorable for microbial growth and the direct addition of preservatives (Hsu et al., 2017; **Lee, Huang, et al., 2023**). The results of this study indicated that the strains exhibited the highest potential for inhibiting the spore germination of OTA-producing fungi (*A. niger*) (Fig. 3 and Table 4).

The changes in pH values before and after fermentation can provide insights into the metabolic characteristics of the fermenting microorganisms. This analysis also aims to determine whether anaerobic fermentation conditions influence pH changes and to compare these changes with the results of spore germination inhibition of *A. niger* BCRC 33485. This comparison will help assess whether pH variation is a key factor in the mechanism of spore germination inhibition. A literature indicated that the antifungal activity of organic acids is significant at pH values below 4.5, suggesting a correlation between the effectiveness of these acids and pH levels (Hunter & Segel, 1973). Recent study has also reported that the production of organic acids during the coffee fermentation process is beneficial in preventing the germination of OTA-producing fungi (**Lee, Huang, et al., 2023**).

The relationship between organic acids and coffee chemistry, as well as the factors influencing coffee acidity, has been extensively discussed (**Lee, Yim, et al., 2023**). Among the various organic acids, citric acid is known for its distinct sour taste, which is commonly associated with citrus fruit flavors. Citric acid, along with malic and quinic acids, constitutes a significant portion of the total acid content in coffee and plays a crucial role in the development of perceived acidity (**Bratthäl et al., 2024**). During the roasting process, citric acid significantly accumulates under light to medium roasting conditions but decreases in concentration as the roasting level increases due to degradation at higher temperatures.

Additionally, acetic acid is produced during the later stages of coffee fermentation and maturation, and roasting conditions may also contribute to an increase in acetic acid content. At low concentrations, acetic acid imparts a refreshing, sweet characteristic to the coffee extract; however, at higher concentrations, it can develop a ferment-like flavor. Malic acid contributes a tart taste and leaves a lingering sensation on the tongue. Although malic acid is present in the coffee fruit itself, roasting has little effect on its content. However, microorganisms during fermentation may play a role in the accumulation of malic acid.

The presence and proportion of various acids in coffee significantly impact its diverse and harmonious flavor profile. The relationship between acidity and the overall flavor profile of coffee is complex and subtle (Rune et al., 2023). However, the influence of roasting on the acid composition aligns with findings from previous studies on roasted coffee (Verardo et al., 2002), indicating that the roasting process has a significant effect on the stability and expression of different acids. Therefore, further investigation into the changes in acid composition during roasting is crucial for understanding the development of coffee flavor. We found that the level of lactic acid was lowered by various fermentation with yeast, the lactic acid metabolism in yeast was investigated (Da Mota et al., 2022). Regarding sensory properties, citric acid contributes to the sourness characteristic of coffee (Rocha et al., 2024).

## 5 Conclusion

Fermentation of fresh coffee cherries, processing, roasting, and brewing into coffee beverages, whether with or without inoculation of microbial fermentation, results in good flavor. This study explores two reusable approaches. The first investigates whether the metabolites from coffee fermentation microorganisms can inhibit the germination of *Aspergillus* fungal spores. The results show that the *Saccharomyces cerevisiae* strain exhibits better inhibitory capacity. Although *Pichia kudriavzevii* showed some inhibitory effects in two groups, the inhibitory effect was not consistent between different groups of the same strain, suggesting variability in individual characteristics. Previous literature has primarily focused on biological control methods to inhibit the growth of microorganisms that pose food safety risks in coffee. This study explores the ability of metabolites, meaning that even without live microorganisms, food safety objectives can still be achieved.

## CRediT authorship contribution statement

**Tsung-Yu Liu:** Methodology, Investigation, Funding acquisition, Conceptualization. **Wei-Hsuan Hsu:** Writing – original draft, Supervision, Software, Resources, Conceptualization. **Bao-Hong Lee:** Writing – review & editing, Writing – original draft, Project administration, Methodology, Investigation.

## Declaration of competing interest

The authors declare that they have no known competing financial interests or personal relationships that could have appeared to influence the work reported in this paper.

## Data Availability

No data was used for the research described in the article.

## References

[bb0005] Abid S., Hassen W., Achour A., Skhiri H., Maaroufi K., Ellouz F., Bacha H. (2003). Ochratoxin A and human chronic nephropathy in Tunisia: Is the situation endemic?. Human and Experimental Toxicology.

[bb0010] Annunziata L., Campana G., De Massis M.R., Aloia R., Scortichini G., Visciano P. (2024). Ochratoxin A in foods and beverages: Dietary exposure and risk assessment. Exposure and Health.

[bb0015] Avallone S., Guiraud J.P., Guyot B., Olguin E., Brillouet J.M. (2001). Fate of mucilage cell wall polysaccharides during coffee fermentation. Journal of Agricultural and Food Chemistry.

[bb0020] Batista L.R., Chalfoun S.M., Prado G., Schwan R.F., Wheals A.E. (2003). Toxigenic fungi associated with processed (green) coffee beans (*Coffea arabica* L.). International Journal of Food Microbiology.

[bb0025] Batista L.R., Chalfoun S.M., Silva C.F., Cirillo M., Varga E.A., Schwan R.F. (2009). Ochratoxin A in coffee beans (*Coffea arabica* L.) processed by dry and wet methods. Food Control.

[bb0030] Bendele A.M., Carlton W.W., Krogh P., Lillehoj E.B. (1985). Ochartoxin A carcinogenesis in the C57BL/6JxC3HF1 mouse. Journal of the National Cancer Institute.

[bb0035] Bratthäl T., Figueira J., Nording M.L. (2024). Influence of divalent cations on the extraction of organic acids in coffee determined by GC-MS and NMR. Heliyon.

[bb0040] Bressani R., Estrada E., Jarquin R. (1972). Composición química y contenido de aminoácidos de la proteína de la pulpa. Turrialba.

[bb0045] Bryden W.L. (2007). Mycotoxins in the food chain: Human health implications. Asia Pacific Journal of Clinical Nutrition.

[bb0050] Bucheli P., Taniwaki M.H. (2002). Research on the origin, and on the impact of post-harvest handling and manufacturing on the presence of ochratoxin A in coffee. Food Additives and Contaminants.

[bb0055] Bui-Klimke T.R., Wu F. (2015). Ochratoxin A and human health risk: A review of the evidence. Critical Reviews in Food Science and Nutrition.

[bib206] Bukhari S.A., Salman M., Numan M., Javed M.R., Zubair M., Mustafa G. (2020). Characterization of antifungal metabolites produced by *Lactobacillus plantarum* and *Lactobacillus coryniformis* isolated from rice rinsed water. Molecular Biology Reports.

[bb0060] Castegnaro M., Canadas D., Vrabcheva T., Petkova-Bocharova T., Chernozemsky I.N., Pfohl-Leszkowicz A. (2006). Balkan endemic nephropathy: Role of ochratoxins A through biomarkers. Molecular Nutrition & Food Research.

[bb0065] Chopra M., Link P., Michels C., Schrenk D. (2010). Characterization of ochratoxin A-induced apoptosis in primary rat hepatocytes. Cell Biology and Toxicology.

[bb0070] Cruz-O’Byrne R., Gambasica N.P., Forero S.A. (2020). Physicochemical, microbiological, and sensory analysis of fermented coffee from Sierra Nevada of Santa Marta, Colombia. Coffee Science.

[bb0075] Da Mota M.C.B., Batista N.N., Dias D.R., Schwan R.F. (2022). Impact of microbial self-induced anaerobiosis fermentation (SIAF) on coffee quality. Food Bioscience.

[bb0080] Evangelista S.R., Miguel M.G.C.P., Silva C.F., Pinheiro A.C.M., Schwan R.F. (2015). Microbiological diversity associated with the spontaneous wet method of coffee fermentation. International Journal of Food Microbiology.

[bb0085] Guzmán-Alvarez R.E., Márquez-Ramos J.G. (2021). Fermentation-processes, benefits and risks. IntechOpen.

[bb0090] Haubeck H.D., Lorkowski G., Kolsch E., Roschenthaler R. (1981). Immunosuppression by ochratoxin A and its prevention by phenylalanine. Applied and Environmental Microbiology.

[bb0095] Hsu W.H., Lai Y.J., Wu S.C. (2017). Effects of the anti-microbial peptide paraxin plus sodium erythorbate dissolved in different gels on the quality of Pacific white shrimp under refrigerated storage. Food Control.

[bb0100] Hunter D.R., Segel I.H. (1973). Effect of weak acids on amino acid transport by Penicillium chrysogenum: Evidence for a proton or charge gradient as the driving force. Journal of Bacteriology.

[bb0105] Krogh P. (1992). Role of ochratoxin in disease causation. Food and Chemical Toxicology.

[bb0110] Lee B.H., Huang C.H., Liu Y.Y., Liou J.S., Hou C.Y., Hsu W.H. (2023). Microbial diversity of anaerobic-fermented coffee and potential for inhibiting ochratoxin-produced *Aspergillus niger*. Foods.

[bb0115] Lee H., Yim J., Lee Y., Lee K.G. (2023). Effect of organic acid-soaking and sonication on the formation of volatile compounds and α-dicarbonyl compounds in Robusta coffee. Ultrasonics Sonochemistry.

[bb0120] Leitão A.L. (2019). Occurrence of ochratoxin a in coffee: Threads and solutions-a mini review. Beverages.

[bb0125] Lima C.M.G., Costa H.R.D., Pagnossa J.P., Rollemberg N.D.C., da Silva J.F., Dalla Nora F.M., Verruck S. (2022). Influence of grains postharvest conditions on mycotoxins occurrence in milk and dairy products. Food Science and Technology.

[bb0130] Mayura K., Reddy R.V., Hayes A.W., Berndt W.O. (1976). Teratogenic and toxic effects of ochratoxin a in rats. Toxicology and Applied Pharmacology.

[bb0135] Murthy P.S., Naidu M.M. (2012). Sustainable management of coffee industry by-products and value addition- a review. Resources, Conservation and Recycling.

[bb0140] Nakasone K.K., Peterson S.W., Jong S.C. (2004). Preservation and distribution of fungal cultures. Biodiversity of Fungi.

[bb0145] Napolitano A., Fogliano V., Tafuri A., Ritieni A. (2007). Natural occurrence of ochratoxin a and antioxidant activities of green and roasted coffees and corresponding byproducts. Journal of Agricultural and Food Chemistry.

[bb0150] Nehad E.A., Farag M.M., Kawther M.S., Abdel-Samed A.K., Naguib K. (2005). Stability of ochratoxin a (OTA) during processing and decaffeination in commercial roasted coffee beans. Food Additives and Contaminants.

[bb0155] Paterson R.R.M., Lima N. (2010). How will climate change affect mycotoxins in food?. Food Research International.

[bb0160] Payne C., Bruce A. (2001). The yeast *Debaryomyces hansenii* as a short-term biological control agent against fungal spoilage of sawn *Pinus sylvestris* timber. Biological Control.

[bb0165] Rocha R.A.R., da Cruz M.A.D., Silva L.C.F., Costa G.X.R., Amaral L.R., Bertarini P.L.L., Santos L.D. (2024). Evaluation of arabica coffee fermentation using machine learning. Foods.

[bb0170] Rune C.J.-B., Giacalone D., Steen I., Duelund L., Munchow M., Clausen M.P. (2023). Acids in brewed coffees: Chemical composition and sensory threshold. Current Research in Food Science.

[bb0175] Shi Y.C., Lai C.Y., Lee B.H., Wu S.C. (2022). The bacterial and fungi microbiota of soy sauce-supplied lactic acid bacteria treated with high-pressure process. Fermentation.

[bb0180] Shi Y.C., Wu S.C., Lin Y., Ching, Zheng Y.J., Huang C.H., Lee B.H. (2024). Development of fermented Atemoya (*Annona cherimola × Annona squamosa*)-Amazake increased intestinal next-generation probiotics. Food Chemistry.

[bb0185] Silva C.F., Batista L.R., Abreu L.M., Dias E.S., Schwan R.F. (2008). Succession of bacterial and fungal communities during natural coffee (*Coffea arabica*) fermentation. Food Microbiology.

[bb0190] Sinnelä M.T., Pawluk A.M., Jin Y.H., Kim D., Mah J.H. (2021). Effect of calcium and manganese supplementation on heat resistance of spores of *Bacillus* species associated with food poisoning, spoilage, and fermentation. Frontiers in Microbiology.

[bb0195] Studer-Rohr I., Dietrich D.R., Schlatter J., Schlatter C. (1995). The occurrence of ochratoxin A in coffee. Food and Chemical Toxicology.

[bb0200] Tsubouchi H., Terada H., Yamamoto K., Hisada K., Sakabe Y. (1988). Ochratoxin A is found in commercial roast coffee. Journal of Agricultural and Food Chemistry.

[bb0205] Verardo G., Cecconi F., Geatti P., Giumanini A.G. (2002). New procedures for determination of acids in coffee extracts, and observations on the development of acidity upon ageing. Analytical and Bioanalytical Chemistry.

